# Production of FMDV virus-like particles by a SUMO fusion protein approach in *Escherichia coli*

**DOI:** 10.1186/1423-0127-16-69

**Published:** 2009-08-11

**Authors:** Chien-Der Lee, Yao-Pei Yan, Shu-Mei Liang, Ting-Fang Wang

**Affiliations:** 1 Institute of Molecular Biology, Academia Sinica, Taipei 11529, Taiwan, Republic of China; 2 Agriculture Biotechnology Research Center, Academia Sinica, Taipei 11529, Taiwan, Republic of China

## Abstract

Virus-like particles (VLPs) are formed by the self-assembly of envelope and/or capsid proteins from many viruses. Some VLPs have been proven successful as vaccines, and others have recently found applications as carriers for foreign antigens or as scaffolds in nanoparticle biotechnology. However, production of VLP was usually impeded due to low water-solubility of recombinant virus capsid proteins. Previous studies revealed that virus capsid and envelope proteins were often posttranslationally modified by SUMO *in vivo*, leading into a hypothesis that SUMO modification might be a common mechanism for virus proteins to retain water-solubility or prevent improper self-aggregation before virus assembly. We then propose a simple approach to produce VLPs of viruses, e.g., foot-and-mouth disease virus (FMDV). An improved SUMO fusion protein system we developed recently was applied to the simultaneous expression of three capsid proteins of FMDV in *E. coli*. The three SUMO fusion proteins formed a stable heterotrimeric complex. Proteolytic removal of SUMO moieties from the ternary complexes resulted in VLPs with size and shape resembling the authentic FMDV. The method described here can also apply to produce capsid/envelope protein complexes or VLPs of other disease-causing viruses.

## Background

Foot-and-mouth disease (FMD) is a severe, highly contagious viral disease of cloven-hoofed animals, such as cattle, pig, sheep, goats and deer, and is the most economically devastating livestock disease in the world. The 2001 outbreak in the UK caused stock losses of more than 12 billion euros. Although animals can be protected against FMD by vaccination with killed FMDV, prophylactic vaccination is still impossible for several reasons. First, the danger that vaccinated animals may become carriers due to residual live virus in the vaccine would make it impossible to maintain an international export trade in meat and livestock. Second, at current prices, the cost of prophylactic vaccination using the killed FMDV is too high. Third, killed virus vaccines are not produced in many FMD-free countries. Unfortunately, the risks of new outbreaks are increasing due to globalization and the increased possibility of bioterrorism. If it were introduced into the United States, which is FMD-free, the disease could cause 100 billions of dollars in losses to the U.S. economy [1]. The development of a cheap and noninfectious subunit or virus-like particle (VLP) vaccine for prophylactic use is therefore a matter of world-wide interest. VLPs consist of protein(s) derived from the capsid of a virus. They are noninfectious because while they resemble the virus from which they were derived, they lack the viral nucleic acids. VLPs used as vaccines are often very effective at eliciting both T cell and B cell immune responses [2,3]. VLPs have also recently found application as as carriers for foreign antigens [2,3] or scaffolds in nanoparticle biotechnology [4].

FMDV is a small RNA virus (27 nm in diameter) of the *Picornaviridae *family (see ). FMDV particles are composed of 60 copies of each of four capsid proteins termed VP1, VP2, VP3 and VP4, which are cleavage products of the P1 capsid precursor polypeptide. During assembly, five protomers, each containing one copy of VP0, VP1, and VP3, assemble into a pentamer, and 12 pentamers associate with a newly transcribed RNA molecule to form a virus particle. Cleavage of VP0 to VP2 and VP4, which is considered to be autocatalytic, is normally observed only upon encapsidation of RNA in mature virions [1]. Attempts have been made to produce subunit vaccines by expressing FMDV capsid protein(s) in *E. coli*. These recombinant capsid proteins exhibited poor water solubility and were administered in vaccine studies either in denatured forms [5-7] or after a tedious refolding procedure [8]. To overcome this technical bottleneck, we recently developed an improved SUMO (Smt3) fusion protein system in *E. coli *to produce several water-soluble virus capsid proteins, including FMDV-VP3 [9]. Here, Smt3 serves not only as a solubility enhancer but also as a protease recognition site. SUMO proteases have the advantage of recognizing the tertiary structure of Smt3, rather than a linear amino acid sequence like other proteases commonly used in the fusion protein approach [10].

Studies have revealed that the capsid and envelope proteins of several viruses could either interact with SUMO or Ubc9 (the SUMO E2 ligation enzyme) or were SUMO modified during virus infection; these viruses including the Tula hantavirus, Epstein-Barr virus, cyto-Megalo virus, Dengue virus, herpes virus and Molony murine leukemia virus [11-14]. Moreover, quantitative SUMO modification of a vaccinia virus protein A40R prevents A40R proteins from self-polymerization and aggregation *in vivo *[15]. We postulated that SUMO modification might be a common mechanism for virus proteins to retain their solubility or to prevent improper aggregation before virus assembly [9]. We believe this hypothesis can be empirically tested by SUMO fusion protein approach, because several sumoylated proteins have been mimic *in vivo *by fusing a SUMO moiety at their N- or C-terminal ends [16-18].

In this report, we first simultaneously expressed SUMO fusion proteins of three FMDV capsid proteins (i.e., VP0, VP1 and VP3) in *E. coli*. These three fusion proteins form a ternary complex. This protein complex was then subjected to cleavage with a home-made SUMO protease to yield a VP0-VP1-VP3 complex. Finally, we showed by EM imaging analysis that the VP0-VP1-VP3 complexes were able to assemble into VLPs *in vitro*.

## Materials and methods

### Plasmid constructions and protein production

**Figure 1:**
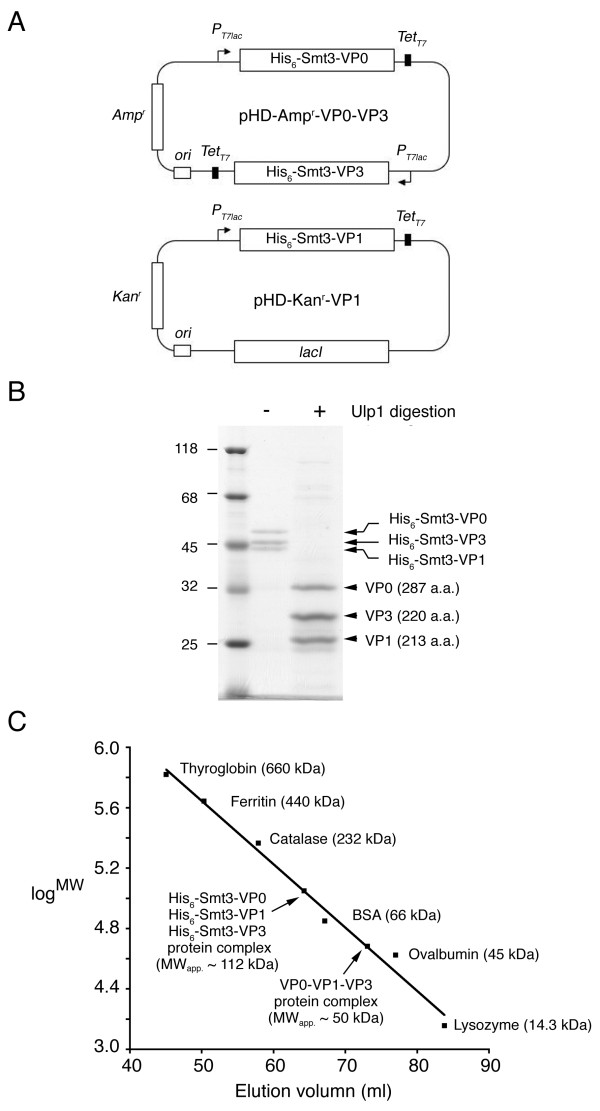
**Production of water-soluble FMDV capsid protein complexes**. (A) Dual SUMO fusion protein expression vectors for simultaneous expression of His_6_-Smt3-VP0, His_6_-Smt3-VP1 and His_6_-Smt3-VP3. The two vectors were transformed into BL21(DE3)-RIL *E. coli *cells, and selected by ampicillin (Amp^r^) and kanamycin resistance (Kan^r^). (B) SDS-PAGE of the purified capsid protein complexes without (left lane) or with (right lane) Ulp1 protease treatment. (C) Gel filtration. Purified capsid protein complexes were layered onto a size exclusion column (HiLoad 16/60 Superdex, GE Healthcare), and eluted at 1.0 ml/min. The elution volume is indicated. Values shown here are the established MW_theo. _of commercial globular protein standards, and the computed MW_app. _of capsid protein complexes from the plot.

An improved SUMO fusion protein expression system was carried out as described before [9]. This system uses an expression vector with *P*_*T7lac *_promoter and Tet_*T*7 _terminator for expressing SUMO fusion proteins. This vector is referred here as pHD-Amp^r^, as it carries an ampicillin resistant gene (Amp^r^). In brief, the cDNA encoding any target protein X is amplified by sticky-end polymerase chain reaction (PCR), then subcloned into SUMO fusion protein vectors using two universal cloning sites, *Sfo*I at the 5' end and *Xho*I at the 3' end. The resulting expression vector allows one to express a His_6_-Smt3-X fusion protein that can be affinity purified on Ni^2+^-resins. The purified fusion protein is then subjected to proteolytic digestion with a home-made SUMO protease, His_6_-Ulp1_403–621_-His_6_. The resulting product X is authentic, because its amino acid sequence is identical to that encoded by the cDNA. We reported before that a pHD-Amp^r^-VP3 vector was successfully applied to produce water-soluble virus capsid authentic FMDV VP3 protein [9]. To simultaneously express three SUMO fusion proteins in the same *E. coli *cell, we first constructed a pHD-Kan^r ^vector by replacing Amp^r ^of the pHD-Amp^r ^vector with a kanamycin resistant gene (Kan^r^). We then constructed pHD-Kan^r^-VP0 and pHD-Kan^r^-VP1 vectors for expressing His_6_-Smt3-VP0 and His_6_-Smt3-VP1 fusion proteins, respectively (Fig. 1A). A DNA fragment containing *P*_*T7lac*_, His_6_-Smt3-VP0 and Tet_*T*7 _was PCR amplified from the pHD-Kan^r^-VP0 vector, and then subcloned into pHD-Amp^r^-VP3 vector to generate a dual fusion protein expression vector, pHD-Amp^r^-VP0-VP3 (Fig. 1A). Both pHD-Amp^r^-VP0-VP3 and pHD-Kan^r^-VP1 vectors were transformed into *BL21(DE3)-RIL E. coli *cells (Stratagen, USA), and selected by ampicillin and kanamycin resistance. Expression, purification and proteolytic cleavage of His_6_-Smt3 fusion proteins were carried out as described before [9].

### Gel filtration

Gel filtration was performed by high-pressure liquid chromatography with a HiLoad 16/60 Superdex sizing column (GE Healthcare) and ran at a flow rate of 1.0 ml/min. The elution profile was followed by a continuous assay of the optical density at 280 nm. Commercially supplied molecular mass standards (including thyroglobulin, ferritin, catalase, bovine serum albumin, ovalbumin and lysozyme) were used to calibrate this column. The average elution position, *K*av, was computed by the equation *K*av = (*Ve *- *V*_0_)/(*Vt *- *V*_0_), where *Ve *and *Vt *represent elution volumes for the molecular mass standards and DTT, respectively. *V*_0_, the void volume, was determined by blue dextran 2000 (GE, Healthcare).

### Electron microscopy

A droplet (4 μl) of purified protein complex was placed for 1 min at room temperature on a copper grid (300 mesh, Pelco, USA) coated with fresh carbon. The excess buffer was then carefully blotted away from the edge of the grid with Whatman #1 filter paper (Whatman Inc., USA). After staining for 4 min with 2.5% uranyl acetate, excess liquid was removed and the samples were air dried at room temperature. Bio-transmission electron microscopy (EM) was performed with a Tecnai G2 Spirit Bio TWIN (FEI Co., Netherlands) using an acceleration voltage of 120 kV. Images were recorded with a slowscan CCD camera (Gatan MultiScan 600) at a resolution of at least 1024 × 1024 pixels.

## Results

Three SUMO fusion proteins (His_6_-Smt3-VP0, His_6_-Smt3-VP1 and His_6_-Smt3-VP3) were simultaneously expressed in the same *E. coli *host cell (Fig. 1A; see Experimental procedures). Their theoretical molecular weights (MW_theo._) are 45,175 Da, 37,057 Da and 37,245 Da, respectively. These three SUMO fusion proteins were all strongly induced by isopropyl β-D-thiogalactoside and were water-soluble. Extracts containing coexpressed SUMO fusion proteins were purified on a Ni^2+^-resin that selectively retains His_6_-tagged polypeptides. Upon examination by SDS-PAGE stained with Coomassie Blue, the eluates predominately contained a protein triplet with identical staining signals (Figure 1B, left lane). The size of the purified fusion proteins was examined by gel filtration, by comparison with globular protein standards. The purified proteins eluted from a size exclusion column at an elution volume expected for a globular protein with apparent MW (MW_app._) of 112 kDa (Figure 1C), very close to the MW_theo. _(119,468 Da) of a 1:1:1 ternary protein complex of these three SUMO fusion proteins. This ternary complex is not due to interactions between His_6_-Smt3 moieties on these fusion proteins. After His_6_-Smt3 moieties were cleaved off from the fusion proteins with SUMO protease, His_6_-Smt3 alone existed as monomers in solution (data not shown). We also found that the His_6_-Smt3-VP1 fusion protein became water-soluble only when it was expressed together with both His_6_-Smt3-VP0 and His_6_-Smt3-VP3 in the same *E. coli *host cell (data not shown). Therefore, this ternary complex likely contains one copy of each of these three SUMO fusion proteins. Since the protomer of the FMDV envelope also contains one copy of each of VP0, VP1 and VP3 [1], our results suggest that the purified fusion proteins are structurally similar to the authentic virus capsid proteins.

**Figure 2:**
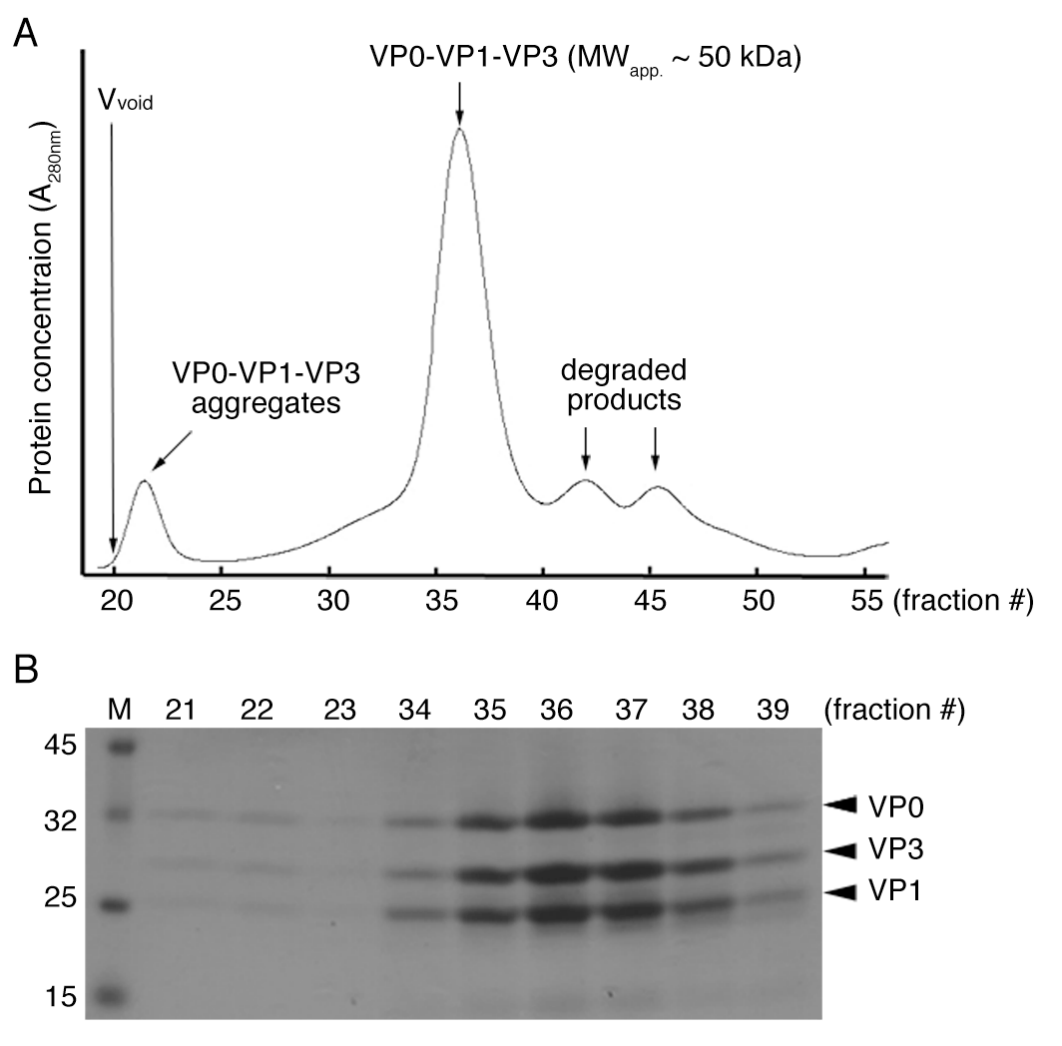
**Purified VP0-VP1-VP3 capsid protein complexes spontaneously formed large protein aggregates**. (A) Gel filtration. Purified capsid protein complexes (Fig. 1C) were reloaded onto a size exclusion column (HiLoad 16/60 Superdex, GE Healthcare), and eluted at 1.0 ml/min. The elution volume is indicated. A significant portion of VP0-VP1-VP3 capsid protein complexes formed large protein aggregates. (B) SDS-PAGE of VP0-VP1-VP3 protein complexes from indicated fractions.

**Figure 3:**
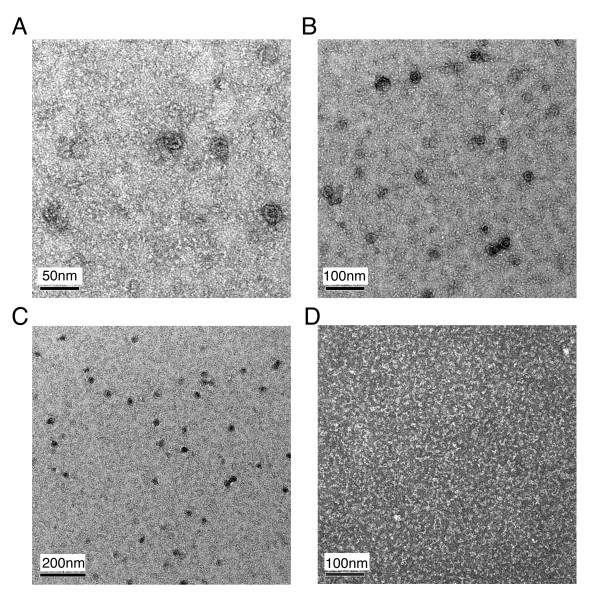
**The VP0-VP1-VP3 protein complexes form round VPLs *in vitro***. Negative-staining electron microscopy images of the purified VP0-VP1-VP3 proteins (A-C) and the His_6_-Smt3-VP0/His_6_-Smt3-VP1/His_6_-Smt3-VP3 ternary complexes (D). Scale bars (in black) are 50, 100 or 200 nm as indicated.

The purified SUMO fusion protein complexes were then treated with SUMO protease to remove the His_6_-Smt3 moieties. The cleavage products contained three water-soluble polypeptides with MW_app. _~32 kDa (VP0), 29 kDa (VP3) and 26 kDa (VP1), respectively (Figure 1B, right lane). Edman degradation was then performed to confirm that the N-termini of these three polypeptides were identical to the expected amino acid sequences of authentic VP0, VP1 and VP3 proteins (data not shown). The MW_theo. _and isoelectric points (*pI*) of these polypeptides are 31747 Da (*pI *= 5.55), 23639 Da (*pI *= 9.66) and 23,815 Da (*pI *= 4.87), respectively. Moreover, these polypeptides migrated together in a size exclusion gel filtration column, and eluted at an elution volume expected for a globular protein with MW_app. _~50 kDa (Figure 1C), indicating that they form a 1:1:1 non-globular complex (MW_theo. _= 79,201 Da). The final yield of purified VP0-VP1-VP3 ternary complexes is ~5 mg of protein per liter of cell culture. It is unlikely that this eluted protein peak contains a mixture of dimmers of each individual protein since His_6_-Smt3-VP1 became water-soluble only when it was co-expressed with both His_6_-Smt3-VP0 and His_6_-Smt3-VP3 in the same *E. coli *host cell. Finally, we found that the purified VP0-VP1-VP3 ternary complexes could assemble into large protein aggregates (Figure 2) which eluted faster than thyroglobin (MW ~660 kDa) in the same size exclusion gel filtration column described in Figure 1C. We then used transmission electron microscopy to examine the extent of VLP assembly by these ternary protein complexes (Fig. 3A–C). Indeed, the VP0-VP1-VP3 complexes formed round VLP aggregates with diameters ~25 nm. A previous EM study had revealed that the size of authentic FMDV is 27 nm [1]. By contrast, the ternary complexes of His_6_-Smt3-VP0, His_6_-Smt3-VP1 and His_6_-Smt3-VP3 formed hardly any round-shaped VLP (Fig. 3D). Taken together, we conclude that VP0-VP1-VP3 complexes are capable of forming VLP *in vitro*, and that the presence of His_6_-Smt3 fusion components impedes VLP assembly between SUMO fusion proteins.

## Discussion

In this study, we have demonstrated for the first time the production of FMDV VLPs in *E. coli*. A bacterial expression system using *E. coli *has numerous advantages over other expression systems, including ease of handling, much lower cost and efficient generation time. The FMDV VLPs so produced may be applicable for prophylactic vaccination or as molecular tools in nanobiotechnology [4]. Finally, viruses within the *Picornaviridae *family show much conservation in capsid protein composition and structural organization, including hepatitis A virus (HAV), the polio virus and the EV71 hand-foot-and-mouth disease virus. Recently, we also have successfully applied the same approach to produce soluble VP0-VP1-VP3 ternary complexes of HAV (CD Lee and Wang TF, unpublished data). Therefore, our method likely will be applicable to produce capsid protein complexes or VLPs of other *Picornaviridae *viruses.

Our results also indicated that addition of SUMO moieties could prevent aggregation of FMDV capsid proteins. After the SUMO moieties were removed from these capsid proteins, they were able to form VLPs with size and shape resembling those of native FMDV viruses. These results are consistent with the hypothesis that SUMO modification not only increases the solubility of capsid proteins but also prevents improper aggregation before virus assembly. It is of interest to further investigate if SUMO modifications indeed control virus assembly *in vivo*. Finally, our study here reveals a simple way to control FMDV VLP assembly *in vitro*, it may be useful in nanoparticle biotechnology.

## Competing interests

The authors declare that they have no competing interests.

## Authors' contributions

CDL and TFW designed the experiments and analyzed the data. TFW wrote the paper and the principle investigator. CDL and YPY carried out the experiments. SML contributed the genes encoding the VP proteins of FMDV and participating in the initiation of the project. All authors read and approved the final manuscript.
